# A Multi-Sensor Stochastic Energy-Based Vibro-Localization Technique with Byzantine Sensor Elimination

**DOI:** 10.3390/s23239309

**Published:** 2023-11-21

**Authors:** Murat Ambarkutuk, Sa’ed Alajlouni, Pablo A. Tarazaga, Paul E. Plassmann

**Affiliations:** 1 The Bradley Department of Electrical and Computer Engineering, Virginia Tech, Blacksburg, VA 24061, USA; murata@vt.edu; 2 Department of Mechatronics Engineering, Faculty of Engineering, The Hashemite University, Zarqa 13133, Jordan; saed@hu.edu.jo; 3 J. Mike Walker ‘66 Department of Mechanical Engineering, Texas A&M University, College Station, TX 77843, USA; ptarazaga@tamu.edu

**Keywords:** occupant localization, structural vibrations, sensor fusion, Byzantine sensor elimination

## Abstract

This paper presents an occupant localization technique that determines the location of individuals in indoor environments by analyzing the structural vibrations of the floor caused by their footsteps. Structural vibration waves are difficult to measure as they are influenced by various factors, including the complex nature of wave propagation in heterogeneous and dispersive media (such as the floor) as well as the inherent noise characteristics of sensors observing the vibration wavefronts. The proposed vibration-based occupant localization technique minimizes the errors that occur during the signal acquisition time. In this process, the likelihood function of each sensor—representing where the occupant likely resides in the environment—is fused to obtain a consensual localization result in a collective manner. In this work, it becomes evident that the above sources of uncertainties can render certain sensors deceptive, commonly referred to as “Byzantines.” Because the ratio of Byzantines among the set sensors defines the success of the collective localization results, this paper introduces a Byzantine sensor elimination (BSE) algorithm to prevent the unreliable information of Byzantine sensors from affecting the location estimations. This algorithm identifies and eliminates sensors that generate erroneous estimates, preventing the influence of these sensors on the overall consensus. To validate and benchmark the proposed technique, a set of previously conducted controlled experiments was employed. The empirical results demonstrate the proposed technique’s significant improvement ($\tilde{3}$0%) over the baseline approach in terms of both accuracy and precision.

## 1. Introduction

Structural-vibration-based occupant localization is a perception methodology where occupants’ locations in an indoor environment are determined by analyzing the floor vibrations due to their footfall patterns. Specifically, this method employs the measurements of accelerometers that are fixed to the floor. Henceforth, the terms vibro-localization and vibro-measurements will refer to such localization techniques and the measurements used in these techniques, respectively. Vibro-localization techniques facilitate a myriad of applications ranging from smart home monitoring and event classification [1,2,3,4,5] to human gait assessment [6,7,8,9,10,11,12] and occupant identification and tracking [13,14,15,16,17,18,19,20].

Energy-based vibro-localization techniques utilize the energy that is inherent in vibro-measurements as a localization feature, since the signal energy serves as a consistent metric for gauging the magnitude of the vibro-measurements [21,22,23,24,25]. Specifically, higher signal amplitudes result in larger energy values registered by the sensors, and vice versa. By employing this notion, energy-based vibro-localization techniques characterize the relationship between the signal energy and the length of the propagation path. Therefore, these techniques offer a simplified approach to occupant localization, thereby reducing the need for exhaustive signal analysis. In this study, the terms signal energy, power, intensity, and strength are used interchangeably despite their nuanced differences.

Dispersion is a natural phenomenon where different frequency components of structural waves propagate at different velocities in the medium, i.e., the floor. This phenomenon has been seen as one of the major contributors to localization errors; hence, substantial scholarly endeavors have been directed toward examining the floor’s dispersive attributes on localization outcomes. These researchers have mitigated the dispersive effects inherent in the floor by isolating narrow frequency bands. These bands, derived via continuous wavelet transformation, remain unaffected by dispersion [26,27,28,29]. Kwon and Agha [26], for instance, presented a successful human activity recognition system utilizing floor vibro-measurements. Their technique employed a feature extraction step based on wavelet packet decomposition coupled with statistical measures to capture the unique characteristics of different activities. The empirical findings emphasized the efficacy of the proposed technique in the precise identification of diverse human activities. Racic et al. [27] presented a technique for the detection and classification of human activities via floor vibrations. They employed an approach involving a combination of wavelet-based feature extraction and a support vector machine classifier to accurately identify different activities, demonstrating the potential of floor vibro-measurements for activity monitoring and recognition in smart environments. Such narrowband filtering essentially compromises the spatial resolution of the localization technique at hand; this challenge, especially in the context of radio-localization, has been discussed in detail (for instance, [30]).

In their study, Mirshekari et al. [31] employed the wavelet transformation to isolate less-dispersion-affected narrowband frequency spectra, crucial for precise time-difference-of-arrival (TDoA) estimation. This innovative multilateration algorithm, unlike conventional methods, does not rely on prior wave velocity knowledge and adapts effectively to different surfaces. It also integrates a sensor elimination approach to enhance vibro-localization accuracy. Selecting the nearest sensors minimizes wave propagation through the floor, mitigating signal attenuation. This improves the signal-to-noise ratio (SNR), reduces the distortion, and enhances the TDoA and location estimations. Localization with four sensors is favored over more, as additional distant sensors introduce attenuation and lower accuracy. Sensor elimination employs TDoA estimations to exclude distant sensors.

The warped frequency transformation technique has been utilized to discern the dispersion curve and to mitigate perturbations attributed to dispersion [32,33]. There exist system-theoretic techniques that characterize the dynamic behavior of the floor via transfer function estimation [34,35,36]. Additionally, the Green’s function has been employed to account for wave reflections and their dispersion [37]. Despite their precision in empirical analyses, these techniques exhibit limitations in their capacity to generalize the complex material properties and boundary conditions inherent in floors.

On the other hand, Bahroun et al. [38] presented their work formalizing the group velocities, i.e., a major component of the signal energy, as a function of the propagation path distance. These promising results paved the way for model-based techniques which tend to explain the wave phenomena from the data. Alajlouni et al. [39], for instance, showed their hypothesis of an energy-decay model (energy logarithmically decays with propagation distance) as a localization model. In their work, Pai et al. [40] analyzed whether a relationship exists between the occupant’s footfall patterns and the measured signal characteristics in an empirical case study. In light of their work, the authors assert that there is no evidence of a monotonic relationship between the amplitude or kurtosis of the measured signal and the propagation distance. Parametric energy-decay models highlight the potential for further improvement [41,42,43].

Along with parametric decay models, Poston et al. [44], in an alternative attempt, moved the localization framework to a probabilistic framework by modeling the probability of detection and false alarm. Alajlouni et al. [41] took a similar approach to localize the occupant by maximizing the sensor likelihood functions given the hypothesis of the sensor’s time domain measurements and the energy-decay model proposed in their earlier work. Wu et al. [42] proposed G-Fall, a device-free fall detection system based on floor vibrations collected using geophone sensors. Their system utilizes hidden Markov models (HMMs) and an energy-based vibro-localization technique, achieving precise and user-independent fall detection with a significant reduction in false alarm rates.

### 1.1. Limitations of the Existing Approaches

One of the inherent challenges limiting the ability and success of vibro-localization techniques is that these techniques are single-shot estimators: each heel-strike and its corresponding vibro-measurement vectors are unique; hence, repeated measurements for a single step are not easily attainable. Therefore, most of the common estimation frameworks cannot be directly employed in such localization systems. This challenge brings about the following limitations in the landscape of vibro-localization techniques.

1. Limitations of ideal sensor models: Accelerometers are not ideal because they tend to introduce random and systematic errors in the vibro-measurement vector during the signal acquisition time. While not trivial, the characterization of errors stemming from signal acquisition is not a central aspect of the existing literature. A complete understanding about localization errors cannot be achieved unless such sensor imperfections are categorically identified and incorporated into vibro-localization frameworks.
2. Limitations in uncertainty quantification: The extent to which measurement errors contribute to localization errors is still unknown. In other words, the sensing errors in vibro-measurements are yet to be tied to the localization errors. It is imperative to account for errors in vibro-measurement vector for a successful localization technique.
3. Limitations in information reliability assessment: Along with the measurement imperfections, a myriad of uncertainty sources drive the success and failure cases of the energy-based vibro-localization techniques, such as reflections, dispersion, etc. To remedy the adverse effects of any unreliable sensor information at a given time, a metric needs to be proposed to measure the reliability of the information that each vibro-measurement vector carries.

### 1.2. Baseline Study and Overview of the Fundamental Differences

In this subsection, we present a comprehensive comparison between the established baseline in vibro-localization, as outlined by Alajlouni and Tarazaga [45], and the proposed technique. The fundamental differences between these two methodologies are summarized in Table 1. The comparison encompasses the key elements of vibro-localization, including the type of localization features measured, the known parameters assumed in both techniques, the calibrated parameters used during offline processing, and the final output produced during online processing. This comparative analysis aims to highlight the enhancements and novel contributions of the proposed technique.

In contrast, our study builds upon and extends this model by considering non-linear factors that may affect the energy–distance relationship. These factors could include environmental characteristics and multi-path effects, which cannot be accounted for in a purely linear model.

To emphasize the contributions of this study, it is essential to contrast our approach with the closest-related work, as encapsulated in [41]. While [41] initiates the exploration of the problem space by deriving probability density functions (PDFs) of the energy of the acquired vibro-measurement vectors, it does so with the simplifying assumption of neglecting the cross-terms as they are zero-mean random variables. Our work diverges fundamentally at this juncture, where we incorporate all terms in our derivation. This inclusion introduces a more comprehensive model that acknowledges the potential influence of cross-terms.

Further diverging from [41], we abandon the assumption that energy measurements $e_{i}$ are independent and identically distributed. This work demonstrates, through Proof 1 and Corollary 1, that the $e_{i}$’s are, in fact, sampled from distinct distributions for each sensor. We substantiate this claim by providing the PDFs and the first two statistical moments for these distributions. This nuanced understanding of the energy measurements’ distribution is pivotal to enhancing the accuracy of vibro-localization techniques, thereby marking a significant stride forward from the state of the art.

### 1.3. Summary of the Contributions

This paper presents an energy-based vibro-localization technique that addresses the sensor imperfections and their effects on the localization results. The proposed technique employs a family of accelerometers placed on a floor to generate multiple vibro-measurement vectors for the same step. Furthermore, the proposed technique employs two corrective steps in the localization time: (i) a comprehensive uncertainty quantification to minimize the effect of the internal errors occurring during the signal acquisition time present in the vibro-measurement vectors; and (ii) an information-theoretic BSE algorithm to address the external sources of uncertainty such as reflections and dispersion. The following points summarize the proposed technique’s contributions.

- **Vibro-localization technique with comprehensive uncertainty quantification** (addressing limitations 1 and 2): The proposed vibro-localization technique employs an explicit error model for each sensor. Therefore, a complete uncertainty quantification of the localization errors due to the measurement errors can be minimized with our technique.
- **Information-theoretic BSE algorithm** (addressing limitation 3): The paper introduces a BSE algorithm. The proposed BSE algorithm divides the sensors into two distinct subsets: the ones that show some consistency among them, and the ones which are divergent in nature. By leveraging a greedy information-theoretic approach, it decides whether a sensor should be placed in the former set, or vice versa. This algorithm guarantees a locally optimal subset of the sensors in minimizing the localization errors.
- **Empirical validation** (addressing limitations 1–3): Data from previously conducted controlled experiments were employed to validate and benchmark the proposed technique. The results demonstrated significant improvements over the baseline [45] approach in terms of both accuracy and precision.
- **Quantification of the empirical precision and accuracy** (addressing limitation 3): This paper employs the results of the empirical validation study to quantify an empirical correlation between the precision and accuracy achieved with the proposed vibro-localization technique. By employing such correlation metrics, we gain better insights about the technique’s performance and failures.

### 1.4. Organization of the Paper

This paper is structured into six distinct sections to provide a comprehensive overview of the research topic.

- The first section, Section 1, serves as the introductory portion of the article. In this section, readers are introduced to the primary problem that the research addresses. Additionally, it offers a review of the existing literature on the subject, ensuring that readers have a foundational understanding of the context and the significance of the problem at hand.
- Following the Introduction, Section 2 delves into the specifics of the proposed technique. This section provides the details of the uncertainty quantification of the localization outcomes due to the errors in the vibro-measurement vectors. A probabilistic technique is employed to quantify the localization uncertainties. Here, we also provide details of the BSE algorithm used in the elimination of Byzantine sensors.
- In the subsequent section, Section 3, the focus is on the controlled experiments that were carried out. This section provides a detailed account of the experimental setup and procedure, laying the groundwork for the results that follow.
- Section 4 presents the results obtained from the experiments. It offers insights into the technique’s performance and reliability, discussing the outcomes in the context of the proposed method’s effectiveness.
- Section 5 encapsulates the primary findings of the research. It presents the conclusions drawn from the study and sheds light on potential avenues for future work. This section serves to summarize the research’s contributions and provide future research directions.

## 2. Method

In this section, the manuscript provides its principal contribution: a new energy-based vibro-localization technique. As an individual traverses the environment, the force exerted by their heel-strikes on the floor induces structural vibration waves within it. These waves propagate through the floor and reach the sensors placed on the floor thereby generating vibro-measurements. Along the propagation path, the vibration wave is deformed and disturbed by various factors that are internal and external to the floor. The proposed technique employs these deformed vibro-measurements of the floor to determine the locations of occupants, despite challenges posed by measurement uncertainties and the potential presence of Byzantine sensors.

Definitions and notation: Consider the problem of estimating a single footstep location $x_{i}\in R^{2}$ that belongs to an occupant located at $x_{true}\in R^{2}$. In this estimation problem, the vibro-measurements $z_{i}\left[k\right]$ that are obtained by the *i*th of *m* sensors placed under a floor between time steps k∈{1,…,n} are employed. Let M≜{1,…,m} be the index set of all the sensors and sensor i∈M be located at $t_{i}$ in a rectangular localization space S. The localization space S is an arbitrary closed shape within which all the sensors and the occupants are contained. Assume, the vibro-measurements $z_{i}\left[k\right]$ are disrupted with measurement error $\zeta_{i}\left[k\right]$, where the true vibro-measurements are $z_{true,i}\left[k\right]$. We derive the relationship between the true vibro-measurements and the sensor’s output as

(1) $$z_{i}\left[k\right]=z_{true,i}\left[k\right]+\zeta_{i}\left[k\right]\;.$$

We often use a shorthand notation to represent these quantities in vector form associated with the time snapshot between 1≤k≤n. Let us define these vectors for the sake of clarity: $z_{i}≜{\left(z_{i}\left[1\right],\ldots ,z_{i}\left[n\right]\right)}^{⊤}\in R^{n}$, $z_{true,i}≜{\left(z_{true,i}\left[1\right],\ldots ,z_{true,i}\left[n\right]\right)}^{⊤}\in R^{n}$ and $\zeta_{i}≜{\left(\zeta_{i}\left[1\right],\ldots ,\zeta_{i}\left[n\right]\right)}^{⊤}\in R^{n}$.

**Figure 1:**
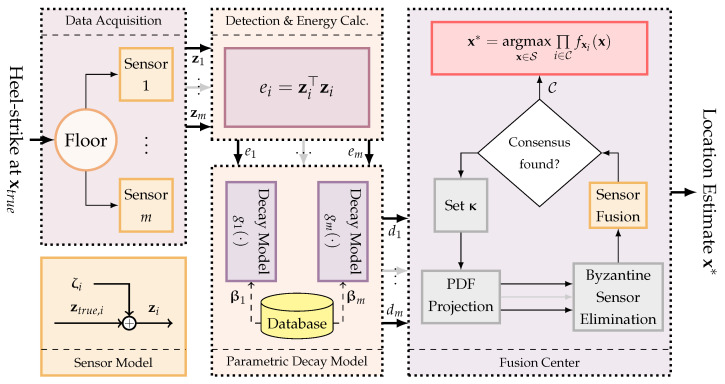
Illustration of the localization methodology proposed in this manuscript. Structural vibrations, resulting from the heel-strikes of an occupant, are detected by *m* accelerometers positioned within the environment. Utilizing the signal energy, denoted as $e_{i}$, the distance $d_{i}$—established between the sensor *i* and the occupant—is estimated. Following this, each sensor’s estimation, represented by a PDF, is projected onto the Cartesian localization space, symbolized as S. The entropies derived from the resultant PDFs play a pivotal role in identifying and subsequently excluding potential Byzantine sensors, employing an iterative sensor fusion approach. Upon achieving a consensus among the sensors, after the exclusion of the Byzantine sensors, the localization process is deemed complete.

Given these definitions, we derive a probabilistic localization framework that makes use of the signal energy of the vibro-measurement vector $z_{i}$ and its PDF with a novel BSE algorithm to benefit from as many sensors as possible in estimating the location vector $x_{true}$. The proposed localization technique employs a parametric energy-decay model to estimate the distance between the sensor and the occupant. Figure 1 provides a graphical representation of the concepts of the proposed localization.

The energy $e_{i}$ of the stochastic vibro-measurement vector $z_{i}$ can be derived by employing Rayleigh’s theorem, as shown in Theorem A1 in Appendix A. The signal energy of a random measurement vector $z_{i}$ is

(2) $$e_{i}={∥z_{i}∥}_{2}^{2}=z_{i}^{⊤}z_{i}\in R_{+}\;.$$

By plugging Equation (1) into Equation (2), we derive the energy as

(3) $$e_{i}={\left(z_{true,i}+\zeta_{i}\right)}^{⊤}\left(z_{true,i}+\zeta_{i}\right)\;.$$

Notice that $e_{true,i}=z_{true,i}^{⊤}z_{true,i}$; therefore, the relationship between the measured energy $e_{i}$ and the energy that was supposed to be registered, $e_{true,i}$, is given by

(4) $$e_{i}=e_{true,i}+\varepsilon_{i}$$

where $\varepsilon_{i}=\zeta_{i}^{⊤}\zeta_{i}+2\zeta_{i}^{⊤}z_{i}$ represents the error in the signal energy.

We assume the disturbance vector $\zeta_{i}$ is an independently and identically distributed normal random vector, i.e., $\zeta_{i}\left[k\right]∼N\left(\mu_{\zeta },\sigma_{\zeta }^{2}\right)$. Therefore, we can show that $z_{i}∼N\left(z_{true,i}+\mu_{\zeta },\sigma_{\zeta }^{2}I\right)$. With this, we present our proposition showing that $f_{E_{i}}(e_{i})$ can be approximated with a normally distributed random variable when the number of samples *n* is sufficiently large.

**Proposition** **1.** *The energy of a stochastic vibro-measurement vector can be approximated with a normally distributed random variable with a mean $\mu_{E}$ and variance $\sigma_{E}^{2}$.*

(5) $$e≃N\left(\mu_{e},\sigma_{e}^{2}\right)\;.$$

The details of this assertion can be seen below.

**Proof.**  If both sides of Equation (3) are divided by the variance $\sigma_{\zeta }^{2}$, we have

$$\frac{e_{i}}{\sigma_{\zeta }^{2}}=\frac{1}{\sigma_{\zeta }^{2}}z_{i}^{⊤}z_{i}\;.$$

Recall $\zeta_{i}\left[k\right]∼N\left(\mu_{\zeta },\sigma_{\zeta }^{2}\right)$; therefore, $\frac{\zeta_{i}\left[k\right]}{\sigma_{\zeta }}∼N\left(\frac{\mu_{\zeta }}{\sigma_{\zeta }},1\right)$. The left-hand side of the equation above is a random variable that is the squared sum of *n* independently and identically distributed normal random variables that have unit variance.

Thus, we can show that $\frac{e_{i}}{\sigma_{\zeta }^{2}}∼\chi_{n}^{\prime 2}\left(\lambda \right)$ follows a noncentral chi-squared distribution with n degrees of freedom and the noncentrality parameter $\lambda =\frac{1}{\sigma_{\zeta }^{2}}{\left(z_{true,i}+\mu_{\zeta }\right)}^{⊤}\left(z_{true,i}+\mu_{\zeta }\right)$ (cf. Theorem A2 in Appendix A).

By invoking Theorem A3 in Appendix A, we derive an approximated PDF of $e_{i}/\sigma_{\zeta }^{2}$ with a normal distribution when the number of time samples is sufficiently large (empirically n>20),

$$\frac{e_{i}}{\sigma_{\zeta }^{2}}\approx N\left(n+\frac{1}{\sigma_{\zeta }^{2}}{\left(z_{true,i}+\mu_{\zeta }\right)}^{⊤}\left(z_{true,i}+\mu_{\zeta }\right),2n+\frac{4}{\sigma_{\zeta }^{2}}{\left(z_{true,i}+\mu_{\zeta }\right)}^{⊤}\left(z_{true,i}+\mu_{\zeta }\right)\right)\;.$$

Finally, the scalability property of the normal distribution can be employed to derive the distribution of the signal energy $e_{i}≃N\left(\mu_{E_{i}},\sigma_{E_{i}}^{2}\right)$, where the mean and variance are characterized as shown below:

(6a) $$\begin{array}{cc} \mu_{E_{i}} & =E\left[e_{i}\right]=\sigma_{\zeta }^{2}n+{\left(z_{true,i}+\mu_{\zeta }\right)}^{⊤}\left(z_{true,i}+\mu_{\zeta }\right)\;, \end{array}$$

and

(6b) $$\begin{array}{cc} \sigma_{E_{i}}^{2} & =\mathrm{Var}\left[e_{i}\right]=2\sigma_{\zeta }^{4}n+4\sigma_{\zeta }^{2}{\left(z_{true,i}+\mu_{\zeta }\right)}^{⊤}\left(z_{true,i}+\mu_{\zeta }\right)\;. \end{array}$$

   □

As a consequence of Proposition 1, we present the next corollary:

**Corollary** **1.** *If the sensor is calibrated such that the sensor bias is negligibly small, i.e., $\mu_{\zeta }≊0$ and the variance in the measurement error is known $\sigma_{\zeta }^{2}$, then the energy distribution can be parameterized with the number of samples n and the unknown true energy $e_{true,i}=z_{true,i}^{⊤}z_{true,i}$ that the sensor was supposed to register:*

$$e_{i}∼f_{E_{i}}\left(e_{i};e_{true,i}\right)\approx N\left(n\sigma_{\zeta }^{2}+e_{true,i},2n\sigma_{\zeta }^{4}+4e_{true,i}\sigma_{\zeta }^{2}\right)=\frac{1}{\sigma_{E_{i}}\sqrt{2\pi }}\exp\left[-\frac{{\left(e_{i}-\mu_{E_{i}}\right)}^{2}}{2\sigma_{E_{i}}^{2}}\right]\;,$$

*where the mean $\mu_{E}$ and variance $\sigma_{E}^{2}$ of the energy distribution are given by*

(7a) $$\begin{array}{cc} \mu_{E_{i}} & =E\left[e_{i}\right]=\sigma_{\zeta }^{2}n+e_{true,i}\;, \end{array}$$

*and*

(7b) $$\begin{array}{cc} \sigma_{E_{i}}^{2} & =\mathrm{Var}\left[e_{i}\right]=2n\sigma_{\zeta }^{4}+4e_{true,i}\sigma_{\zeta }^{2}\;. \end{array}$$

### 2.1. Parametric Energy Decay and Localization Framework

**Figure 2:**
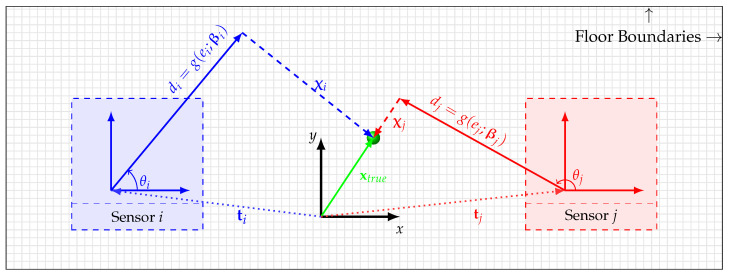
This figure visualizes some key variables frequently used in the paper. The blue and red boxes represent sensor *i* and sensor *j* which reside at $t_{i}$ and $t_{j}$, respectively. When an occupant excites the floor with their footstep, which occurs at $x_{true}$, *m* accelerometers first estimate $d_{i}\forall 1,\ldots ,m$. Therefore, the estimated location vector of the occupant location by sensor *i* can be seen as the vector summation of its location vector $t_{i}$ and the estimated $d_{i}$ for some $\theta_{i}$.

In this work, we exploit the notion that the signal energy decreases as the structural vibration wavefronts propagate along a path. Based on this concept, we propose a localization function $h_{i}:{\left(e_{i},\theta_{i}\right)}^{⊤}\mapsto x_{i}$ that maps the signal energy $e_{i}$ concerning the vibro-measurement vector $z_{i}$ and the directionality of the occupant $\theta_{i}$ to a location vector $x_{i}\in S$. The localization function $h_{i}\left(e_{i},\theta_{i};\beta_{i}\right)$ can be represented as

(8) $$x_{i}=h_{i}\left(e_{i},\theta_{i};\beta_{i}\right)=t_{i}+g_{i}\left(e_{i};\beta_{i}\right)\left[\begin{array}{c} \cos\theta_{i}\\ \sin\theta_{i} \end{array}\right]\;,$$

where $\beta_{i}$ represents a known calibration vector of a parametric energy-decay model $g\left(e_{i};\beta_{i}\right):e_{i}\mapsto d_{i}$ that maps the energy $e_{i}$ to the distance $d_{i}$. The calibration vectors $\beta_{i}$, ∀i∈M, are obtained in a pre-deployment calibration step. A graphical representation of Equation (8) is shown in Figure 2. It should be noted that the parametric energy-decay model g(·) is assumed to be a monotonically decreasing function for some positive energy measurement $e_{i}\in R_{+}$; therefore, it is bijective and its inverse exists. In this work, we employed a curve that represents the relationship between the energy of the vibro-measurement vectors and the propagation distance as a power curve. Formally, this curve can be shown below, $d_{i}=\beta_{0,i}\;e_{i}^{\beta_{1,i}}$, where the calibration vector is given by $\beta_{i}={\left(\beta_{0,i},\beta_{1,i}\right)}^{⊤}$.

In this framework, the occupant location with respect to the *i*th sensor is parameterized with its representation in a polar coordinate system centered at sensor location $t_{i}$. As an accelerometer by itself provides only ranging information, i.e., the distance between the occupant and itself, we assume the directionality component $\theta_{i}$ is completely unknown. Therefore, we can simply model it with a random variable that follows a uniform distribution between the range (0,2π), which is denoted as $\theta_{i}∼U\left(0,2\pi \right)$.

The defining properties of the location estimate $x_{i}$ can be obtained by studying its PDF $f_{X_{i}}(x_{i})$. The derivation of the PDF $f_{X_{i}}(x_{i})$, given the PDF of the signal energy $f_{E_{i}}(e_{i})$, is a straightforward application of the density transformation theorem shown in Theorem A4 in Appendix A. The following section provides the signal model with which the signal energy $e_{i}$ can be computed from the vibro-measurement vector $z_{i}$.

### 2.2. Derivation of the pdf for Location Estimation

Given the localization function $h_{i}\left(e_{i},\theta_{i};\beta_{i}\right)$ that maps ${\left(e_{i},\theta_{i}\right)}^{⊤}$ to $x_{i}$, we can derive the PDF of the location estimate $x_{i}$ using the density transformation theorem, as presented in Theorem A4 in Appendix A,

(9a) $$\begin{array}{cc} f_{X_{i}}\left(x_{i};e_{true,i},\beta_{i}\right) & =f_{E_{i},\theta_{i}}\left(g_{i}^{-1}\left(∥x_{i}-t_{i}∥\right),\theta_{i};e_{true,i},\beta_{i}\right)\left|\det J_{h_{i}}^{-1}\left(x_{i};\beta_{i}\right)\right|\;, \end{array}$$

because $E_{i}$ and $\theta_{i}$ are independent from each other:

(9b) $$\begin{array}{cc} & =f_{E_{i}}\left(g_{i}^{-1}\left(∥x_{i}-t_{i}∥\right);e_{true,i},\beta_{i}\right)f_{\theta_{i}}(\theta_{i})\left|\det J_{h_{i}}^{-1}\left(x_{i};\beta_{i}\right)\right|\;, \end{array}$$

where $f_{\theta_{i}}(\theta_{i})=\frac{1}{2\pi }$ and $f_{E_{i}}\left(e;e_{true,i},\beta_{i}\right)=\frac{1}{\sigma_{E}\sqrt{2\pi }}\exp\left[-\frac{{\left(e-\mu_{E}\right)}^{2}}{2\sigma_{E}^{2}}\right]$. Therefore,

(9c) $$\begin{array}{cc} f_{X_{i}}\left(x_{i};e_{true,i},\beta_{i}\right) & =\frac{1}{\sigma_{E}{\left(2\pi \right)}^{\frac{3}{2}}}\exp\left[-\frac{{\left(g_{i}^{-1}\left(∥x_{i}-t_{i}∥\right)-\mu_{E}\right)}^{2}}{2\sigma_{E}^{2}}\right]\left|\det J_{h_{i}}^{-1}\left(x_{i};\beta_{i}\right)\right|\;, \end{array}$$

where $J_{h_{i}}^{-1}\left(x_{i};\beta_{i}\right)$ denotes the Jacobian matrix of the inverse localization function $h_{i}^{-1}\left(\cdot \right)$ evaluated at $x_{i}$.

The definition of the Jacobian matrix $J_{h_{i}}^{-1}\left(x_{i}\right)$ is given as

(10) $$J_{h_{i}}^{-1}\left(x\right)=\left[\begin{array}{cc} \frac{\partial g_{i}^{-1}}{\partial x}\left({∥x-t_{i}∥}_{2};\beta_{i}\right) & \frac{\partial g_{i}^{-1}}{\partial y}\left({∥x-t_{i}∥}_{2};\beta_{i}\right)\\ \frac{\partial ∠\left(x-t_{i}\right)}{\partial x} & \frac{\partial ∠\left(x-t_{i}\right)}{\partial y} \end{array}\right]\;,$$

where

$$\det J_{h_{i}}^{-1}\left(x\right)=\frac{1}{∥x-t_{i}∥}\frac{\partial g_{i}^{-1}}{\partial x}\left(∥x-t_{i}∥;\beta_{i}\right)\;.$$

By plugging Equation (10) into Equation (9), we derive the PDF fully:

(11) $$\Rightarrow f_{X_{i}}\left(x_{i};e_{true,i},\beta_{i}\right)=\frac{\left|\frac{\partial g_{i}^{-1}}{\partial x_{i}}\left(∥x_{i}-t_{i}∥;\beta_{i}\right)\right|}{∥x_{i}-t_{i}∥\sigma_{E}{\left(2\pi \right)}^{\frac{3}{2}}}\exp\left[-\frac{{\left(g_{i}^{-1}\left(∥x_{i}-t_{i}∥\right)-\mu_{E}\right)}^{2}}{2\sigma_{E}^{2}}\right]\;.$$

Equation (11) shows the PDFs $f_{X_{i}}\left(\cdot \right)$ assign a probability value for an occupant located at vector $x_{i}$ given the inverse of the parametric decay function g(·) and sensor location $t_{i}$. This equation is one of the fundamental contributions of this study. Notice the term in the denominator, i.e., $∥x_{i}-t_{i}∥$, in Equation (11), results in an inverse relationship between the probability values and the distance between the sensor and the impact location.

### 2.3. Sensor Fusion

Given the PDFs $f_{X_{i}}\left(x_{i};e_{true,i},\beta_{i}\right)$ for all sensors indexed by i∈M, we aim to find a joint PDF that represents the collective localization outcome using the vibro-measurements of *m* sensors. As all the sensors are independent of each other, we can represent the joint PDF as given below:

(12) $$f_{X_{1},\ldots ,X_{m}}\left(x_{1},\ldots ,x_{m};e_{true,1},\ldots ,e_{true,m},\beta_{1},\ldots ,\beta_{m}\right)=\prod_{i=1}^{m}f_{X_{i}}\left(x_{i};e_{true,i},\beta_{i}\right)\;.$$

Let $\kappa_{i}=\frac{e_{true,i}}{e_{i}}$ be an independent variable denoting the ratio between the unknown true energy $e_{true,i}$ and measured signal energy $e_{i}$. Notice that $e_{i}=e_{true,i}+\epsilon_{i}$; thus, $\kappa_{i}=1+\Delta$ for small Δ. Given this definition, we can reparameterize the joint PDF which forms the basis for the sensor fusion algorithm used in this paper,

(13) $$f_{X_{1},\ldots ,X_{m}}\left(x_{1},\ldots ,x_{m};\kappa_{1},\ldots ,\kappa_{m},\beta_{1},\ldots ,\beta_{m}\right)=\prod_{i=1}^{m}f_{X_{i}}\left(x_{i};\kappa_{i},\beta_{i}\right)\;.$$

### 2.4. Byzantine Sensor Elimination

**Figure 3:**
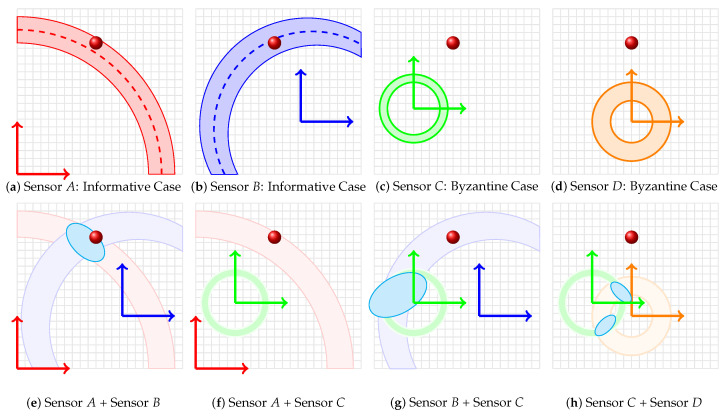
The figure displays eight labeled images (**a**–**h**) in two rows. The first row depicts individual sensor PDFs: (**a**) Sensor *A* with a sharp peak showing high precision; (**b**) Sensor *B* with a broader curve showing accuracy and lower precision; (**c**) Sensor *C*, a Byzantine sensor with an offset sharp peak; and (**d**) Sensor *D* with a flat curve indicating low accuracy and precision. The second row illustrates fusion results: (**e**) a unimodal curve from sensors *A* and *B* showing enhanced precision; (**f**) a uniform distribution from sensors *A* and *C* indicating discord; (**g**) an offset peak from sensors *B* and *C* suggesting an alternative location hypothesis; and (**h**) a bimodal distribution from sensors *C* and *D* with peaks deviating from the true value. The figure highlights the challenges of fusing data from diverse sensors, especially with Byzantine influences.

Byzantine sensors, as illustrated in Figure 3, are those that provide misleading or incorrect data, often deviating from the true value or introducing conflicting information into a sensor network. In the figure, sensors *A* and *B* are examples of informative sensors (shown in images (a) and (b)) while *C* and *D* are instances of Byzantine sensors (images (c) and (d)). The PDFs of the Byzantine sensors have sharp peaks, indicating high precision, but they show offsets from the true value, revealing their low accuracy. When such a sensor’s data are fused with data from other sensors, it can significantly distort the resulting joint likelihood, leading to erroneous conclusions or alternative hypotheses about the occupant’s location.

The second row of the figure provides insights into the effects of fusing data from a Byzantine sensor with informative ones. For instance, image (f) showcases the fusion result of an informative Sensor *A* with the Byzantine Sensor *C*. The resulting uniform distribution across the localization space highlights the lack of consensus between the two, emphasizing the detrimental impact of the Byzantine sensor on the fusion process. Similarly, image (g) depicts the fusion of Sensor *B* with Sensor *C*, producing an offset peak that suggests an alternative hypothesis about the occupant’s location. To ensure the reliability of a sensor network, it is crucial to identify and eliminate such Byzantine influences. By observing the fusion results and identifying distributions that deviate from expected patterns or the true value, one can iteratively pinpoint and remove Byzantine sensors, enhancing the overall accuracy and trustworthiness of the network.

In vibro-localization systems, there exist many factors that can easily render a sensor as a Byzantine. For instance, the parametric energy-decay model assumes the wavefronts detected by an accelerometer travel in a direct path from the step location to the sensor observing the vibration phenomenon. However, this assumption seldom holds for indoor environments as the wavefronts may be reflected by various objects or boundaries that exist in the environment. To address and circumvent these undesired scenarios, we introduce an algorithm that identifies a consensus-forming subset of sensors within M to counteract the influence of Byzantine sensors.

During the initialization phase of the information-theoretic BSE algorithm, a comprehensive computation is carried out to determine all conceivable pairwise joint PDFs and their corresponding entropies. Subsequently, an initial consensus set is established using the sensor pair (i,j) that produces the maximum entropy after fusion from a distinct pair of the index set M. Formally, we represent this initiation phase as given below:

(14) $$C=\left\{i,j|\underset{i,j}{\mathrm{argmax}}E\left[-\log f_{x_{i},x_{j}}\left(x\right)\right]\right\}$$

Furthermore, we represent the joint PDF encapsulating the existing consensus at any point in time, denoted as $f_{C}\left(x\right)$, as follows:

(15) $$f_{C}\left(x\right)≜\prod_{\forall i\in C}f_{X_{i}}\left(x;\kappa_{i},\beta_{i}\right)$$

Throughout each iterative phase, a candidate sensor, denoted as *k* (which is distinct from the pair (i,j)), is selected from the set M. This sensor undergoes an evaluation against the prevailing consensus set C, achieved by fusing its PDF with the consensus PDF $f_{C}\left(x\right)$. A subsequent determination is predicated upon the entropy, or more precisely, the surprisal of the resulting hypothesis with respect to the current consensus. Should the integration of sensor *k* not attenuate the joint PDF to a uniform distribution (as exemplified in case (f) in Figure 3) or not decrease the average entropy, it is incorporated into the consensus set. Otherwise, sensor *k* is classified as Byzantine. Finally, the above procedure undergoes an iterative repetition with varying vectors of $\kappa ={\left(\kappa_{1},\ldots ,\kappa_{m}\right)}^{⊤}$. This iteration progresses in the direction of the gradient of $f_{C}\left(x\right)$ calculated with respect to κ vector. The process persists until a local maximum in the entropy landscape, corresponding to an (possibly local) optimal consensus, is identified. The proposed BSE algorithm, coupled with the vibro-localization technique, is outlined in Algorithm 1.

At an initial glance, readers might find similarities between the algorithm above and the RANSAC algorithm [46], an acronym for random sample consensus; however, they are fundamentally distinct. RANSAC is an algorithm employed in fields such as computer vision and computational geometry to robustly estimate model parameters, even in the presence of outliers. In contrast, the information-theoretic BSE algorithm is tailored for sensor networks to counteract Byzantine sensors using information-theoretic strategies. While both algorithms seek to establish reliability (or robustness) and consensus (or agreement) within datasets, their approaches, and primary use cases are notably distinct. A side-by-side comparison of their characteristics is presented in Table 2.
**Algorithm 1:** Vibro-localization using the Information-Theoretic BSE Algorithm1: **procedure**
BSE(f,M,S) 2:     $h^{*}\leftarrow -\infty$ 3:     **for all** (i,j)∈M×M, i≠j
**do**    ▹ Initialization of the consensus set 4:         $h_{i,j}\leftarrow entropy\left(fuse\left(f_{x_{i}},f_{x_{j}}\right),S\right)$ 5:         **if** $h_{i,j}\leq h^{*}$ **then** 6:            $h^{*},C\leftarrow h_{i,j},\left\{i,j\right\}$ 7:         **end if** 8:     **end for** 9:     **for all** i∈M∖C
**do**       ▹ Attempt to expand the consensus set 10:         $h_{i}\leftarrow entropy\left(fuse\left(f_{C},f_{x_{i}}\right),S\right)$ 11:         **if** $h_{i}\geq h^{*}$ **then** 12:            $h^{*},C\leftarrow h_{i},C\cup \left\{i\right\}$ 13:         **end if** 14:     **end for** 15:     **return** C 16: **end procedure** 17: $\kappa \leftarrow 1_{m\times 1}$ 18: **while**
$∥\nabla \kappa ∥\leq thr$
**do**                 ▹ Gradient Descent 19:     κ+=∇κ              ▹ Update κ in the gradient direction 20:     $f_{x_{1}},\ldots ,f_{x_{m}}\leftarrow density-projection\left(e_{1},\ldots ,e_{m},\kappa \right)$ 21:     $C\leftarrow BSE(f_{x_{1}},\ldots ,f_{x_{m}},M,S)$ 22:     $f_{C}\leftarrow FUSE\left(f_{x_{i}}\;\forall i\in C\right)$ 23:     $x^{*}={\mathrm{argmax}}_{x\in S}f_{C}\left(x\right)$ 24: **end while**


## 3. Experiments

The proposed technique’s effectiveness and performance were assessed through a series of controlled experiments, the details of which are outlined in this section.

### 3.1. Experimental Setup

**Figure 4:**
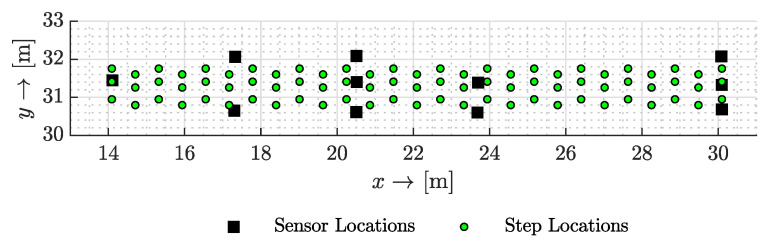
The testbed used in the controlled experiments. The green circles represent the unique step locations while the black squares mark the sensor locations used in the experiments.

To evaluate our vibro-localization approach, we employed the empirical data from a set of controlled experiments in a corridor situated on the fourth floor of Goodwin Hall, an operational building on Virginia Tech’s campus. In these experiments, two participants traversed a pre-defined 16-meter path. Figure 4 represents the step locations constituting the traversed path—represented with green circles (∘)—as well as the sensor locations—represented with black squares (*■*)—overlayed. We derived our reference points from these investigations and utilized identical experimental data as in [41,45]. The corridor’s concrete floor housed the sensors, which were attached to uniform steel mounts welded to the flanges of the structural I-beams beneath. In this study, the experimental testbed utilized is embedded within the structural framework of the building. Due to this integration, a photograph of the testbed would not substantially add to the understanding of the setup, as it predominantly features standard structural components of the building. The crucial aspects of our setup are its configuration and the placement of the sensors and equipment, which are more effectively conveyed through the schematic representation provided in Figure 4. Eleven PCB Piezotronics model 352B accelerometers, detecting dynamic out-of-plane acceleration within the frequency range of (2, 10,000) Hz and with an average sensitivity of 1000 millivolts per *g* (where g=9.8 m/s^2^), recorded the structural vibrations. These devices captured data from 162 steps taken by each participant, amounting to a total of 324 steps. The data collection was facilitated by VTI Instruments EMX-4250 digital signal analyzer cards, connected to the accelerometers via coaxial cables and equipped with anti-aliasing filters and a high-precision 24-bit analog-to-digital converter. The accelerometer data were sampled at a rate of 1024 Hz. For a comprehensive insight into the experimental design, readers are directed to the foundational study [45].

**Data and model validity:** The preliminary study gathered vibration data during low-activity periods, ensuring minimal movement in the vicinity of the instrumented corridor. The data revealed that the sensors’ noise profiles were normally distributed with zero mean and consistent variance. Thus, the signal model in Equation (1) aptly represents the vibration measurements. Given this observation, we confidently state that the energy-related random variables, represented as $e_{i}$ for i∈M, align with the experimental findings.

**Figure 5:**
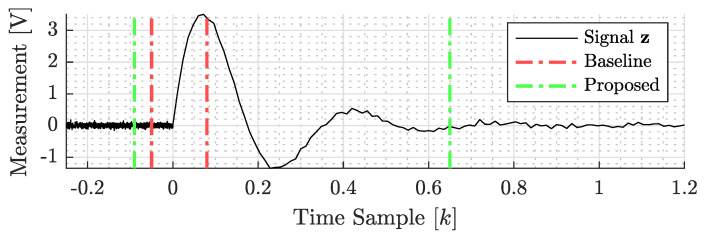
The differences between signal (step) detection algorithms employed by the baseline and proposed techniques. The black line (**—**) represents the noisy measurements of a second-order system. The green dash-dotted line (**— -**) represents the proposed “relaxed” detection results employed in this study. On the other hand, the red dash-dotted line (**— -**) represents the signal detection algorithm employed by the baseline study.

**Signal detection problem:** A primary distinction exists between our implementation of the baseline and the original work presented in the publication [45]: the signal detection algorithm. The baseline study, in its methodology, adopted a tight-window approach. This approach was characterized by the identification of the first time instance that the signal broke the SNR envelope and the peak of the signal. In this study, on the other hand, we used a stochastic signal detection algorithm to denote the time steps in which the signal magnitude broke the noise floor and eventually dissipated below the noise floor. To contrast our method, the detection algorithm employed in this study took a more flexible stance: instead of strictly searching for the time instance, we signal peaks, our algorithm was designed to be more lenient. It permitted “silent” periods, which are intervals without significant signal activity, both before and after the vibration. This choice of algorithm led to a notable difference in the signal energy values when compared to the baseline study. Figure 5 graphically demonstrates the difference between the signal detection algorithm employed by the baseline and the proposed technique with an impulse response curve of an underdamped second-order system. The black line represents the elements of the “noisy” vibro-measurement vector. The dashed red and green lines represent the results of the baseline and proposed detection algorithm, respectively. This distinction in approach not only highlights the variability in signal processing techniques but also underscores the potential impact of these choices on the final results and interpretations.

### 3.2. Implementation

In the course of our data processing, we discretized the localization space S. This discretization was achieved by segmenting it into a total of 270,000 grid cells, specifically arranged in a 300×900 configuration. By evaluating the PDFs only at the center of each grid cell, we avoided the intricate surface integrals and achieved greater computational efficiency.

The calibration vectors $\beta_{i}$ are obtained in an offline processing step, where the signal energy and their known distance measurements are used to fit the parametric decay models, denoted as $g_{i}\left(e_{i};\beta_{i}\right)$. These measurements can be obtained by exciting the floor, for instance, by hitting it with a hammer, at known locations. This process facilitates the accurate calibration of the system by correlating the known physical impacts at specific points with the resulting signal characteristics. In this work, we only used the first 27 steps of Occupant-1’s data to obtain these calibration vectors for all sensors.

In the online processing, when a footstep is detected, the signal energy of the vibration measurements are used to minimize the log-transformed loss function Equation (15) by adjusting the value of vector $\kappa ={\left(\kappa_{1},\ldots ,\kappa_{m}\right)}^{⊤}$ in the direction of its gradient. Consequently, the information-theoretic BSE algorithm is employed in each iteration of gradient descent to discard Byzantine sensors. The gradient descent steps are repeated until the algorithm converges to a solution and forms a consensus among the sensors.

To evaluate the proposed technique, we employed a combinatorial study to analyze the effect of the number of sensors used on the localization metrics. Specifically, we employed all the possible combinations of m={2,…,11} with the given sensor configuration in the experimental data. This yields ncases=∑i=21111i=2036 cases to evaluate for each step and occupant. In other words, each step is re-evaluated 2036 times, yielding 329832 (2036×162) data points for each occupant. Therefore, in our analysis, we are able to provide different defining statistical characteristics of the localization error. This approach also enables us to remedy various uncertainty sources such as the effects of sensor placement, and differences in propagation paths while enriching the results independent of the individual sensor performance. The source code of this study is available on request.

## 4. Results

This section presents the empirical outcomes derived from our proposed vibro-localization technique. These empirical results benchmark the efficacy of our approach in terms of accuracy and precision. By analyzing these findings in detail, we aim to provide a deeper look into the technique’s performance under various conditions and scenarios.

**Figure 6:**
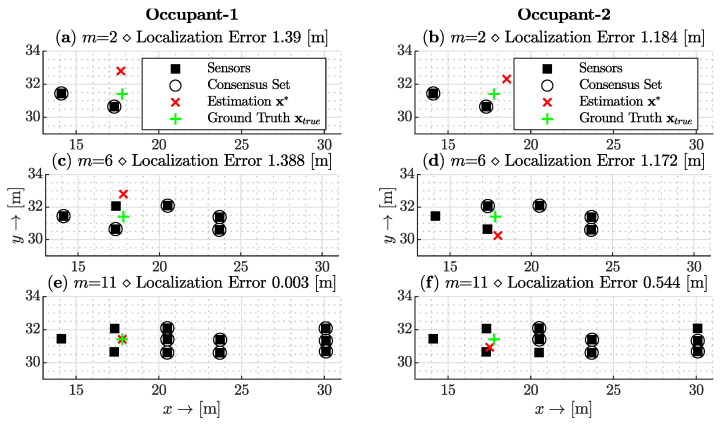
Localization outcomes for two distinct occupants using varying sensor counts (m=2,6,11). The left column represents the first occupant’s result set and the right, the second occupant’s result set. Square markers indicate sensor locations, circles denote non-Byzantine sensors, while green pluses and red crosses symbolize the ground truth and estimated locations, respectively. Errors for configurations (**a**–**e**) show progressive refinement with increased sensors, highlighting the algorithm’s adaptability and precision. **Left:** An illustrative result of the 1st occupant’s data. **Right:** An illustrative result of the 2nd occupant’s data.

In Figure 6, three representative sets (out of 659664 cases) of outcomes are depicted, each corresponding to a different number of sensors (m=2,6,11) employed for the localization of identical step data for two occupants. The left column presents results for the first occupant, while the right column pertains to the second occupant. The sensor locations within this figure are marked by square markers (*■*), and sensors deemed non-Byzantine by the algorithm are highlighted with circular markers (∘). The green plus (+) and red cross (×) markers, respectively, represent the ground truth $x_{\mathrm{true}}$ and the estimated location vector $x^{*}$. In the scenarios labeled (a) for the first occupant and (b) for the second, utilizing two sensors, the norm of the localization errors were 1.39 and 1.1784 meters, respectively. Both sensors were considered as the consensus set due to the lack of alternative sensor choices. As the sensor count increased to six, as shown in labels (c) for the first occupant and (d) for the second, the observed errors were 1.39 for the first occupant and 1.172 meters for the second occupant. However, the first occupant’s results did not show significant improvement with the additional sensors. In these cases, the initial sensors were adaptively substituted with new sensors for consensus. With a further increase to eleven sensors, as indicated in labels (e) and (f), the localization errors reduced to 0.3 centimeters for the first occupant and 54.4 centimeters for the second, both accompanied by an updated consensus set.

**Figure 7:**
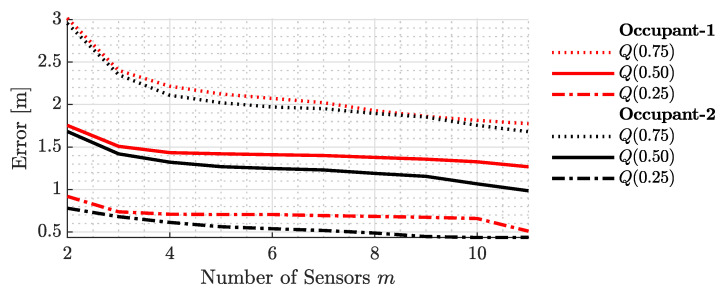
Quartile analysis of sample localization errors against the number of sensors before the proposed BSE algorithm was employed. The plot showcases a consistent reduction in errors across all quartiles with an increasing number of sensors, highlighting improved consistency in both best- and worst-case scenarios.

**Figure 8:**
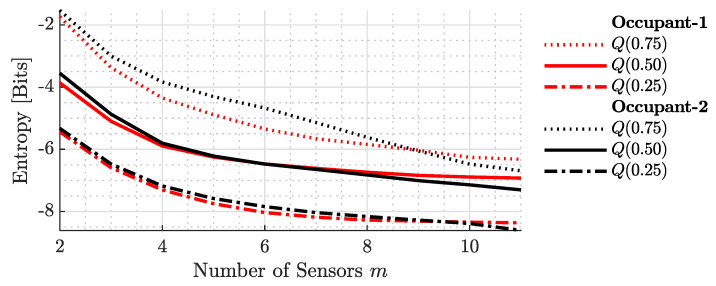
Entropy-based precision of the localization system for varying sensor counts. Red and black lines differentiate data for the first and second occupants. The figure underscores the reduced uncertainty with more sensors, highlighting the enhanced precision across all quartiles.

To evaluate the influence of the sensor count on the localization outcomes, especially in terms of accuracy and precision, the quantile function, represented as x=Q(p), of the localization error was plotted against the number of sensors, as illustrated in Figure 7 and Figure 8. This function yields the error value *x* for a given probability *p*, satisfying the condition $P\left(X\leq x\right)=p$. In essence, it serves as the inverse of the cumulative distribution function (CDF) for the random variable *x*. For clarity, Q(0.5) corresponds to the median of the localization error across varying sensor counts.

In Figure 7, the quartiles of the sample localization errors—the first (25th percentile), second (50th percentile or median), and third (75th percentile)—are plotted against the number of sensors available in the localization system. In other words, the number of sensors listed in the figure represents the sensor count before the proposed BSE algorithm eliminates a subset from the sensor pool. The data for the first and second occupants are differentiated by red and black colors, respectively. For the first occupant, the solid (–), dash-dotted (– -), and dotted (· ·) red lines represent the respective quartiles of the localization error. For the second occupant, the solid (–), dash-dotted (– -), and dotted (· ·) black lines serve the same purpose. The figure indicates a reduction in the localization error with an increasing number of sensors. This trend is consistent across all quartiles. Notably, as more sensors are introduced, the disparity between the first and third quartiles diminishes, highlighting enhanced accuracy in both the optimal and suboptimal conditions.

As is evident from Table 3, a consistent trend across both occupants and all quartiles is seen: as the number of sensors increases, the localization error (measured in all metrics) decreases. For instance, the median error for the first occupant decreases from 2.4481 with two sensors to 1.7931 meters with eleven sensors in the proposed technique (see Table 3). Similarly, for the second occupant, it reduces from 2.3852 to 1.6147 meters for the same number of sensors. The standard deviation, representing the error variability, also shows a steady decrease, an evidence of a growth in consistency, as more sensors are employed. This reduction in error and variability is a clear indication of enhanced accuracy and reliability in the localization process.

Figure 8 presents the precision of the entire localization system in terms of entropy, or the surprisal, encoded in the joint PDFs. Akin to the previous figure, the data for the first and second occupants are differentiated by red and black colors, and the same styling is used to represent the same quartiles. The figure distinctly shows a decrease in uncertainty as the number of sensors grows. This trend is consistent across all quartiles. Significantly, with the addition of more sensors, the gap between the first and third quartiles narrows, indicating enhanced precision in both the best- and worst-case scenarios.

**Figure 9:**
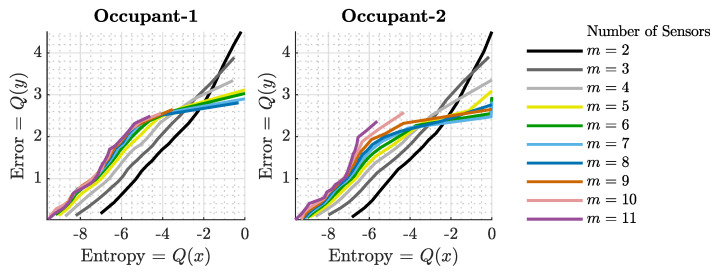
Quantile–quantile plot between the precision and accuracy metrics observed in the experimental data. The figure provides evidence for the correlation between precision and accuracy for varying numbers of sensors.

Figure 9 synthesizes the insights derived from Figure 7 and Figure 8, demonstrating a discernible correlation between the accuracy and precision metrics obtained with the proposed localization technique. The figure demonstrates that enhancements in precision are parallel with improvements in accuracy. This trend can be observed for both of the occupants, even with different numbers of sensors used in the localization system. This trend, as can be seen in the figure, can be described as quasi-linear curves, where the range of the lines differs with the number of sensors used in the technique. Also, it can be seen in the figure that sub-meter localization accuracy is viable even with two sensors if the sensors yield a certain level of measurement precision. On the other hand, for the higher number of sensors, this goal is more attainable as their curves span more in the sub-meter region. Furthermore, note that the magnitude of the improvement in accuracy with the improved precision differs for different numbers of sensors employed in the system. In other words, more desirable results become prominent when more sensors are used.

**Figure 10:**
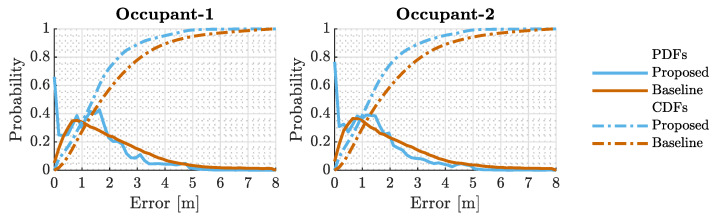
Empirical PDFs and CDFs of normed localization errors derived from location estimates for both occupants. Solid lines represent the empirical PDFs, with blue and brown indicating the proposed and baseline techniques, respectively. Dash-dotted lines depict the empirical CDFs. The plots demonstrate that the proposed technique generally results in lower localization errors compared to the baseline.

The empirical PDFs and empirical CDFs are non-parametric tools employed to analyze the distribution of data points in a sample without assuming an inherent distribution. The empirical PDFs provide a histogram-like representation, highlighting the relative frequencies of various data values, while the empirical CDFs capture the cumulative probability for each value. Figure 10 depicts the empirical PDF, represented with solid lines, and CDF, represented with dash-dotted lines, of the normed localization error derived from location estimates for both occupants’ data. The plots on the left and right represent these curves of the first and second occupants’ data, respectively. The solid blue and brown curves represent the empirical PDF of the proposed and baseline techniques while the dash-dotted curves represent the empirical CDF. As can be seen in the figures, the proposed technique shows relatively higher accumulations in lower regions in the error axis than the baseline suggesting the error characteristics of the proposed technique will more likely fall on the smaller regions than the baseline. Another way to present this observation is through the empirical CDFs. For instance, a major takeaway from the empirical CDF curves is that 80% of the errors of the proposed and baseline techniques are equal to or less than 2.29 and 3.10 meters for both occupants, respectively.

Table 3 tabulates the overall landscape of the resulting error characteristics of the proposed technique and the baseline by providing a statistical analysis comparing the performance of the two localization methods. Various descriptive statistical metrics such as the mean, standard deviation, median, root mean square (RMS), minimum, and maximum values, all expressed in meters, are presented. The results span varying numbers of sensors, from 2 to 11, and are differentiated for two distinct occupants.

For the first occupant, the proposed method consistently outperforms the baseline across all metrics. The weighted average mean localization error for the proposed method is 1.58 meters, a notable improvement from the baseline’s 2.31 meters. Similarly, for the second occupant, the proposed method achieves a weighted average mean error of 1.48 meters, significantly lower than the baseline’s 2.28 meters. Also, one interesting finding from this result set is that the proposed localization technique can achieve sub-meter localization accuracy and precision when enough sensors are employed (cf. std. dev. of Occupant-1 and Occupant-2 with 10 and 11 sensors; cf. median localization error of Occupant-2 with 11 sensors). This table underlines the enhanced accuracy and precision of the proposed vibro-localization technique over the baseline for various sensor configurations and both occupants.

**Figure 11:**
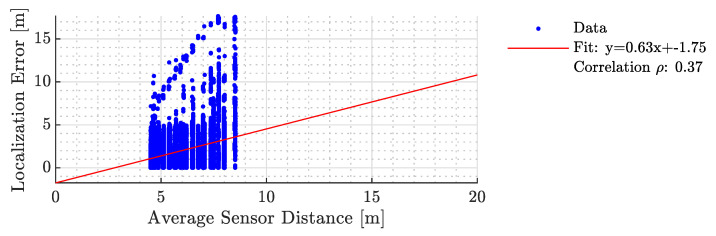
The error characteristics of the proposed method as a function of the average sensor distance when all sensors were considered.

**Figure 12:**
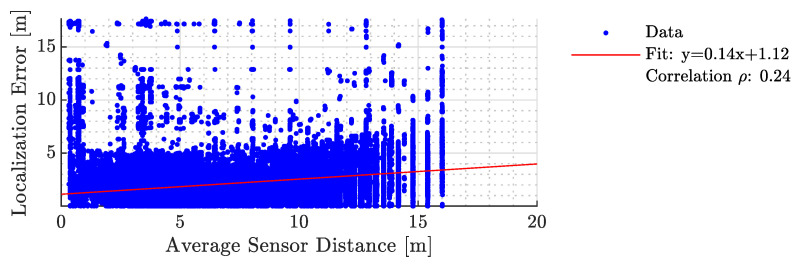
The error characteristics of the proposed method as a function of the average sensor distance when a subset of the sensors were considered.

In Figure 11 and Figure 12, we analyze the relationship between average sensor distance and localization error for two scenarios: considering all sensors and after the BSE algorithm is applied. We used regression analysis to understand the error behavior, with the slope indicating the error increase as occupants move farther from sensors. The correlation between the localization error and the sensor distance assesses the technique’s effectiveness in mitigating systemic errors. An ideal localization technique should minimize these slopes and correlations. Table 4 compares our results with [31].

## 5. Conclusions and Future Work

In this study, we proposed a novel vibro-localization technique that can address two types of uncertainty sources: (i) uncertainties due to sensor imperfections, and (ii) uncertainties due to complexities in wave propagation. To achieve minimum localization errors, the proposed technique coupled with an information-theoretic BSE algorithm employs an uncertainty quantification on the error contributions to the sensors. In this way, the proposed technique minimizes the effect of the errors present in the vibro-measurement technique as well as other sources of uncertainties mentioned above.

In order to benchmark the proposed method, we employed a set of previously conducted controlled experiments. The essence of this validation study was to gauge the efficacy and performance of our proposed technique, especially when contrasted against an existing methodology in the literature. In the experimental setup, which consisted of two participants traversing a 16-meter path, the structural vibrations captured by the closest eleven accelerometers among a much bigger family of accelerometers available in the environment were used. The proposed technique provided significant improvements in almost all localization metrics while best-case and worst-case scenarios became less extreme. The proposed localization technique coupled with the proposed BSE algorithm yielded a 31.47% decrease in the mean localization error.

In this study, we established a consistent empirical relationship between two key metrics of localization systems: accuracy and precision. Although a localization system’s accuracy may not be known post-deployment, we can always determine its precision from the estimates it provides. Therefore, the empirical relationship between these two metrics may be employed to estimate the system’s accuracy without recalibrating it. Although the link between accuracy and precision might be inferential, it has important consequences for the use of these systems in practice.

We have used this correlation to devise a method for estimating the system’s accuracy during operation without the need for additional calibration. Notably, this correlation holds true across different users, which suggests that our findings are robust and widely applicable. Additionally, our results offer a standardized approach to designing experiments. With the established correlation, the number of sensors required for an experiment can be decided based on the desired accuracy and the sensor precision.

Delving deeper into the results, it became evident that the flexibility in our approach, which allowed for silent periods in the signal, could reduce the strong emphasis on signal processing and signal detection steps in real-world scenarios. In other words, a relaxed window around the vibro-measurement vector, which signifies when the vibro-measurements break the noise floor and when it dies down, should be enough for the proposed algorithm to accurately localize an occupant. This is a convenient improvement, as in the employment of such localization systems the event detection problem constitutes a major drawback.

The advancements made in vibro-localization techniques, as presented in this study, open up several promising avenues for further exploration. One potential direction is the integration of global optimization techniques to enhance the accuracy and robustness of localization. By leveraging such techniques, we could refine the estimation process, ensuring that the solution converges to a global minimum, thereby minimizing localization errors. Additionally, a joint solution approach that simultaneously addresses the location estimation problem and the BSE problem could be explored. Such a holistic approach would ensure that the system not only accurately determines the location but also effectively handles unreliable sensor data in a unified framework. This could further streamline the process and potentially lead to real-time localization capabilities with higher reliability.

## Figures and Tables

**Table 1:** Comparative overview of baseline and proposed vibro-localization techniques. This table illustrates the key differences in localization features, known and calibrated parameters, and output between the baseline technique, as per Alajlouni and Tarazaga [45], and the proposed technique.

	Baseline Technique [45]	Proposed Technique
Localization Feature (Measured)	Energy measurements $e_{1},\ldots ,e_{m}$	Energy measurements $e_{1},\ldots ,e_{m}$
Known Parameters (A Priori)	Sensor locations $t_{1},\ldots ,t_{m}$	Sensor locations $t_{1},\ldots ,t_{m}$
Calibrated Parameters (Offline Processing)	None	Sensor noise profile: $\mu_{\zeta },\sigma_{\zeta }$ Calibration vectors $\beta_{1},\ldots ,\beta_{m}$
Output (Online Processing)	Location estimate: $\hat{x}$	Location estimate: $x^{*}$ Consensus set: C and its distribution: $f_{C}\left(x\right)$

**Table 2:** Comparison between RANSAC [31] and information-theoretic BSE algorithm.

	RANSAC [46]	Mirshekari et al. [31]	Information-Theoretic BSE
Primary Use	Estimating parameters of mathematical models in the presence of outliers, predominantly in computer vision.	Elimination of far-away sensors in an adaptive multilateration technique of a vibro-localization system.	Elimination of Byzantine sensors in sensor networks of vibro-localization systems by using information theory.
Methodology	Works by randomly selecting subsets of data and identifying the model with the highest consensus.	Identifying distant sensors in TDoA estimations to avoid bias in multilateration algorithm.	Derives a consensus among sensors based on entropies of likelihoods to emphasize robustness against malicious sensors.
Input Data Type	Points	Time-domain measurements	PDFs

**Table 3:** Comparison of baseline and proposed methods for different numbers of sensors and cases for two occupants. The table presents statistical measures such as mean, standard deviation, median, root mean square (RMS), minimum, and maximum values in meters. Baseline algorithm is adapted from [45].

				Mean (m)		Std. Dev. (m)		Median (m)		RMS (m)		Min (m)		Max (m)	
ine		**# Sensors**	**# Cases**	**Baseline**	**Proposed**	**Baseline**	**Proposed**	**Baseline**	**Proposed**	**Baseline**	**Proposed**	**Baseline**	**Proposed**	**Baseline**	**Proposed**
ine	**Occupant-1**	2	55	3.6888	2.3173	3.3372	2.3253	2.4481	1.7542	4.9742	3.2827	0.0107	0.0011	16.0242	17.5661
		3	165	2.8971	1.8467	2.6042	1.7687	1.9609	1.5082	3.8955	2.5570	0.0092	0.0011	16.0090	17.5661
		4	330	2.5066	1.6502	2.1398	1.4102	1.9153	1.4338	3.2957	2.1707	0.0103	0.0011	14.9141	17.4761
		5	462	2.2905	1.5606	1.8090	1.2039	1.8043	1.4203	2.9187	1.9710	0.0039	0.0011	14.1410	17.4761
		6	462	2.1500	1.5195	1.5744	1.1127	1.7895	1.4097	2.6648	1.8833	0.0026	0.0011	13.2300	11.8762
		7	330	2.0547	1.4868	1.4026	1.0717	1.7807	1.4001	2.4877	1.8328	0.0129	0.0011	12.4402	11.8762
		8	165	1.9854	1.4516	1.2778	1.0440	1.7613	1.3780	2.3611	1.7880	0.0090	0.0011	11.7862	8.3292
		9	55	1.9322	1.4110	1.1878	1.0173	1.7596	1.3571	2.2681	1.7395	0.0249	0.0011	10.5963	5.0333
		10	11	1.8894	1.3714	1.1227	0.9975	1.7415	1.3262	2.1977	1.6956	0.0151	0.0034	9.3303	4.8963
		11	1	1.8545	1.3066	1.0758	0.9818	1.7931	1.2675	2.1423	1.6320	0.1764	0.0034	7.4640	4.6415
ine		**Weighted Average**		2.3056	**1.58**	1.7853	**1.2521**	1.8401	**1.4272**	2.9200	**2.0212**	0.0078	**0.0011**	**13.6703**	14.1558
ine	**Occupant-2**	2	55	3.7099	2.1653	3.3677	2.0995	2.3852	1.6825	5.0103	3.0159	0.0177	0.0010	16.0251	17.6579
		3	165	2.9058	1.7971	2.6500	1.7054	1.9605	1.4196	3.9327	2.4775	0.0017	0.0010	16.0101	16.9322
		4	330	2.4965	1.5820	2.1846	1.4151	1.8451	1.3217	3.3174	2.1225	0.0055	0.0010	14.0716	16.7308
		5	462	2.2650	1.4633	1.8475	1.2276	1.7665	1.2689	2.9229	1.9101	0.0062	0.0010	13.0168	16.7308
		6	462	2.1130	1.4073	1.6066	1.1290	1.7101	1.2475	2.6544	1.8041	0.0026	0.0010	12.3208	16.7308
		7	330	2.0078	1.3721	1.4331	1.0874	1.7021	1.2302	2.4667	1.7507	0.0093	0.0011	11.3596	9.0267
		8	165	1.9322	1.3314	1.3078	1.0590	1.6537	1.1898	2.3332	1.7012	0.0106	0.0011	10.8745	5.8133
		9	55	1.8731	1.2880	1.2215	1.0230	1.6466	1.1549	2.2362	1.6448	0.0074	0.0011	8.7195	5.0686
		10	11	1.8261	1.2356	1.1608	0.9749	1.6713	1.0666	2.1636	1.5736	0.0324	0.0011	6.9466	4.9669
		11	1	1.7871	1.1716	1.1195	0.9224	1.6147	0.9852	2.1069	1.4884	0.1071	0.0011	5.8988	3.8890
ine		**Weighted Average**		2.2771	**1.4843**	1.8217	**1.2545**	1.7755	**1.2790**	2.9194	**1.9444**	0.0063	**0.0010**	**12.7591**	14.2538

**Table 4:** A systemic comparison between the results of work presented in [31] and the proposed localization technique. SE stands for sensor elimination. Desired values are marked with bold face.

	Mirshekari et al. [31]		Proposed	
ine	Without SE	With SE	Without SE	With SE
Slope	1.44	0.56	**0.63**	**0.14**
Intercept	**−2.3**	**−0.56**	1.75	1.12
Correlation Coefficient	0.82	**0.24**	**0.37**	**0.24**

## Data Availability

Data are contained within the article.

## Abbreviations

The following abbreviations are used in this manuscript:

BSE	Byzantine sensor elimination
CDF	Cumulative distribution function
HMM	Hidden Markov model
PDF	Probability density function
SNR	Signal-to-noise ratio
TDoA	Time-difference-of-arrival

## Appendix A

**Theorem** **A1** (Rayleigh’s signal energy theorem). *Rayleigh’s energy theorem states that the energy of a time-domain-continuous and deterministic signal y(t) is the integral of the square of the magnitude of the signal. Equivalently, the energy of the same signal can be obtained from its frequency-domain representation Y(ω).*

(A1) $$e=\int_{-\infty }^{\infty }{\left|y(t)\right|}^{2}\;dt=\int_{-\infty }^{\infty }{\left|Y(\omega )\right|}^{2}\;d\omega$$

*Equivalently, the signal energy of a discrete time signal y[k] is given by*

(A2)
$$e=\sum_{k=-\infty }^{\infty }{\left|y[k]\right|}^{2}=\sum_{k=-\infty }^{\infty }{\left|Y(\omega_{k})\right|}^{2}$$

**Theorem** **A2.**
*The squared sum of n nonstandard normally distributed random variables with unit variance follows the noncentral chi-squared distribution.*

*Let $\left(X_{1},\ldots ,X_{n}\right)$ be normally distributed independent normal random variables with means $\mu_{i}\neq 0$ and unit variances $\sigma_{i}^{2}=1$ for i={1,…,n}. Let Y be the squared sum of the random variables $X_{i}$, as shown in the equation below.*

(A3)
$$Y=\sum_{i=1}^{n}X_{i}^{2}∼\chi_{n}^{\prime 2}\left(\lambda \right)$$

*The resulting random variable Y is said to follow the noncentral chi-squared distribution, denoted $\chi_{n}^{\prime 2}\left(\lambda \right)$. In the short hand representation $\chi_{n}^{\prime 2}\left(\lambda \right)$, n represents the number of random variables used in the summation and is called the degrees of freedom of the distribution, while $\lambda =\sum_{i=1}^{n}\mu_{i}^{2}$ represents the noncentrality parameter.*

**Theorem** **A3.**
*The noncentral chi-squared distribution converges to a normal distribution when n or λ is large.*

(A4)
$$Y∼\chi_{n}^{\prime 2}\left(\lambda \right)≃N\left(n+\lambda ,2n+4\lambda \right)$$

**Theorem** **A4** (Density transformation theorem). *The density of functions of random variables can be calculated with the density transformation theorem. This theorem is essentially an extension of the integration by substitution method.*
*Let $X={\left(X_{1},\ldots X_{m}\right)}^{⊤}\in R^{m}$ be an m-dimensional multivariate random variable with a joint CDF of $F_{X}\left(x\right)=P\left(X\leq x\right)$ and joint PDF of $=\frac{\partial }{\partial x_{1}}\cdots \frac{\partial }{\partial x_{m}}F_{X}\left(x\right)$. Assume $Y={\left(Y_{1},\ldots Y_{n}\right)}^{⊤}\in R^{n}$ is another multivariate random variable defined as a function of X, $Y={\left(g_{1}\left(X\right)\ldots g_{n}\left(X\right)\right)}^{⊤}=G\left(X\right)$, where $g_{i}:\left(X_{1},\ldots ,X_{m}\right)\subset R^{m}\mapsto Y_{i}\subset R$ is an invertible multivariate continuous function.*

*By virtue of the relation between PDF, we can show:*

(A5a)
$$\begin{array}{cc} \int_{Y_{1}}\cdots \int_{Y_{n}} & f_{Y}\left(y\right)\;dy_{1}\ldots \;dy_{n}=\int_{X_{1}}\cdots \int_{X_{m}}f_{X}\left(x\right)\;\;dx_{1}\ldots \;dx_{m}=1 \end{array}$$

*Thus, the PDF of a random variable* Y *can be obtained as given below:*

(A5b)
$$\begin{array}{cc} f_{Y}\left(y\right) & =\frac{\partial }{\partial y_{1}}\cdots \frac{\partial }{\partial y_{n}}\int_{X_{1}}\cdots \int_{X_{m}}f_{X}\left(x\right)\;dx_{1}\ldots \;dx_{m} \end{array}$$

*On the other hand, the CDF of random variable Y can be shown as*

(A6a)
$$\;\begin{array}{cc} F_{Y}\left(y\right) & =P\left(Y\leq y\right)=P\left(X\leq G^{-1}\left(y\right)\right) \end{array}$$

(A6b)
$$\;\begin{array}{cc} F_{Y}\left(y\right) & =\int_{s}^{y}f_{Y}\left(y\right)\;\;dy=\int_{G^{-1}\left(s\right)}^{G^{-1}\left(y\right)}f_{X}\left(x\right)\;\;dx \end{array}$$

*where $s={\left(\sup X_{1},\ldots ,\sup X_{m}\right)}^{⊤}$.*

*Now, substitute $x=G^{-1}\left(u\right)$ in the integral on the right-hand side, i.e., $x=G^{-1}\left(u\right)$ and $\frac{\;du}{\;dx}=G^{\prime }\left(x\right)$. Due to the inverse function theorem, we also have $\frac{\;dx}{\;du}=\frac{\;dG^{-1}}{\;du}$. Consequently,*

(A7a)
$$\begin{array}{c} \int_{s}^{y}f_{Y}\left(y\right)\;\;dy=\int_{s}^{y}f_{X}\left(G^{-1}\left(u\right)\right)\;\frac{\;dG^{-1}}{\;du}\;du \end{array}$$

*Taking the derivative of both sides with respect to y, we have*

(A7b)
$$\begin{array}{cc} f_{Y}\left(y\right) & =f_{X}\left(G^{-1}\left(y\right)\right)\;\frac{\;dG^{-1}}{\;dy} \end{array}$$

(A7c)
$$\;\begin{array}{cc} \; & =f_{X}\left(G^{-1}\left(y\right)\right)\left|\det J_{G^{-1}}\left(y\right)\right| \end{array}$$

*where $J_{G^{-1}}\left(y\right)$ denotes the Jacobian matrix of the inverse function $G^{-1}$ evaluated at y. Due to the implication of the inverse function theorem and the properties of the determinant operator, we have $\left|\det J_{G^{-1}}\left(y\right)\right|=\left|\det\mathrm{inv}J_{G}\left(y\right)\right|=\frac{1}{\left|\det J_{G}\left(x\right)\right|}$, assuming $\det J_{G}\left(x\right)\neq 0$, i.e., G(x) is continuously differentiable.*

(A7d)
$$f_{Y}\left(y\right)=\frac{f_{X}\left(G^{-1}\left(y\right)\right)}{\left|\det J_{G}\left(x\right)\right|}$$
